# An exploration of primary care healthcare professionals’ understanding of pain and pain management following a brief pain science education

**DOI:** 10.1186/s12909-022-03265-2

**Published:** 2022-03-28

**Authors:** Jagjit Mankelow, Cormac G. Ryan, Paul W. Green, Paul C. Taylor, Denis Martin

**Affiliations:** 1 School of Health and Life Sciences, Centuria Building, Teesside University, Middlesbrough, Tees Valley TS1 3BX England; 2 Linthorpe Surgery, 378 Linthorpe Road, Middlesbrough, TS5 6HA England; 3 School of Social Sciences, Humanities and Law Education and Social Work, Teesside University Middlesbrough, Middlesbrough, Tees Valley TS1 3BX England; 4 NIHR Applied Research Collaboration for the North East and North Cumbria, Newcastle Upon Tyne, England

**Keywords:** Pain education, GPs, Nurse practitioners, Mixed-methods

## Abstract

**Background:**

Persistent pain is a leading cause of disability worldwide yet implementation of clinical guidelines that recommend a biopsychosocial approach remains a challenge in clinical practise. Limited pain understanding amongst clinicians may be partly responsible for this.

**Purpose of the study:**

1) Qualitatively explore the experience of receiving PSE, understanding of PSE and operationalisation of PSE-related principles in routine clinical practice. 2) Quantitatively explore pain knowledge, attitudes, and behaviours of general practitioners (GPs) and nurse practitioners (NPs) before and after pain science education (PSE).

**Methods:**

An exploratory, single-site, mixed-methods study in north-east England. Fifteen NPs/GPs completed questionnaires and a case-vignette before and after a 70-min face-to-face PSE lecture. Qualitative data were thematically analysed from two focus groups after the intervention.

**Results:**

Clinicians’ relatively high prior levels of knowledge, attitudes, and behaviour were similar after PSE. Qualitative themes described facilitation of self-reflection on pain management behaviours, and difficulties in operationalising PSE principles in practise including: limited patient rapport; short appointment times; patients’ passive and often oppositional biomedical treatment expectations; and clinicians’ lack of readily understandable language to communicate with patients.

**Conclusion:**

The findings highlight the value of PSE perceived by these clinicians who were already favourably inclined towards biopsychosocial pain management. They sought more resources for their personal learning and for communication with patients. Even with such favourable disposition, the practicalities and environment of clinical practice impeded the operationalisation of PSE-related principles.

**Trial registration:**

This study was prospectively registered at ClinicalTrials.Gov (NCT04587596) in October 2020.

**Supplementary Information:**

The online version contains supplementary material available at 10.1186/s12909-022-03265-2.

## Current research questions

- What impact does a brief pain science education intervention have on knowledge, attitudes and behaviours in healthcare professionals at a GP surgery?
- What is the experience of receiving PSE and how does it affect operationalisation of the concepts?

## What is already known?

- Pain management is frequently based on the biomedical model, rather than the guideline-consistent biopsychosocial model.
- Short pain science education interventions have been shown to be effective for improving students' and patients' pain knowledge, attitudes and behaviours.

## Main messages

- Clinicians with high prior levels of knowledge, attitudes and intention to treat in a guideline consistent way still value pain science education.
- The operationalisation of guideline consistent pain management is difficult due to the practicalities and environment of busy clinical practices.
- Barriers to guideline consistent pain management include patient expectation and difficulties conveying the evidence-base to patients.

## Background

Pain is the most prevalent condition predisposing to disability worldwide [1]. Biological, psychological and socio-environmental factors all have a role in the manifestation of pain [2]. Thus, a biopsychosocial approach encompassing assessment and management of all three of these contributors to pain is necessary for comprehensive pain management. However healthcare professionals (HCPs) are inclined towards a more biomedical approach to pain management than biopsychosocial approaches [3].

An over reliance upon the biomedical approach to pain has been shown to be less successful, cost more and potentially it exacerbates pain and healthcare utilisation [4]. It entails excessive imaging, medication over-prescription and surgeries with poor outcomes, all of which are contrary to guidelines. Recent guidelines for assessment and management of chronic pain advise judicious use and restraint in the prescription of medication, imaging and other passive interventions [5].

Patient perspectives on the barriers to effective pain management include HCP lack of ‘interest and empathy’; lack of GP specialised knowledge in pain management; lack of communication between HCPs; short consultation times with GPs and an absence of a multidisciplinary approach [6]. A holistic biopsychosocial approach to care is sought by patients [6].

Pain science education (PSE) is an evidence-based means of conveying pain education to patients, HCPs and students [7, 8]. The central principle is that pain is not necessarily linked to tissue damage, but indicates a need to protect the body from perceived or real danger [9]. It presents the scientific evidence behind pain and immune, neurological and endocrine system adaptations using metaphors and imagery and illustrates the way in which in addition to biological factors, psychosocial factors also have a role in the manifestation of pain. RCTs in student HCPs show that brief PSE interventions improve pain related knowledge, beliefs and clinical decision making. Furthermore PSE can enable HCPs to reconceptualise pain and foster empathy [10].

Thus, PSE could be a useful brief intervention for time-limited clinicians as a form of continuing professional development (CPD) to facilitate an evidence-based approach to pain management. However, in the same way that PSE should be delivered in combination with other interventions within a clinical setting for patients, ideally PSE would be part of a broader package of CPD for health care professionals to enhance their approach to pain management [9]. The aims of this exploratory mixed-methods study were to explore the effect of a one-off PSE delivery to HCP staff at a GP surgery (or doctors’ office) in north-east England (1) qualitatively identifying their understanding of pain following the education, and the impact of it upon their daily practise and (2) quantitatively by measuring the impact upon pain related knowledge, attitudes and behaviours.

## Methods

### Design

In this, exploratory, single-site, mixed-methods study, primary care professionals’ pain attitudes, knowledge and behaviour data were collected using two questionnaires and a case vignette pre and post-delivery of PSE. Additionally, data about the HCPs’ profession and age were collected. Qualitative data collected during two focus groups post intervention was thematically analysed. The philosophical approach applied to this mixed-methods study was pragmatism wherein the quantitative data and qualitative data provide context to one another particularly if dissonance between the quantitative and qualitative data should arise [11].

### Setting

Clinicians from a GP surgery in north-east England were invited to participate in the study during their usual monthly CPD session. GPs and NPs were invited by email from the practice manager to participate in the study. Data was collected in 2019. The GP surgery served approximately 19,300 patients. The patient age demographic for this GP surgery was similar to the national average. It is in the second lowest decile for the deprivation measurement scale. The GP surgery scored 2 on the deprivation measurement scale of 1–10 (one being the most deprived) [12].

### Intervention

Participants were invited to attend a 70 minute PSE lecture delivered by an advanced practice physiotherapist at the surgery (PG). The lecture was a mostly didactic, powerpoint presentation, centred around material from the book *Explain Pain* [13]. The intervention included metaphors and brief interactive activities intended to facilitate a contemporary pain science, biopsychosocial-centred understanding of persistent pain. PG has 10 years of experience in the delivery of PSE to patients in different settings. At the time of the intervention PG had been working at the GP surgery for two years.

### Outcome measures

Participants completed pre and post-intervention outcome measures to assess pain related knowledge and attitudes. Outcome measures included: 1) the 12-item Revised Neurophysiology of Pain Questionnaire (RNPQ) to measure pain knowledge, and 2) the 13-item Health Care Providers’ Pain and Impairment Relationship Scale (HC-PAIRS) to measure attitudes towards chronic pain [14, 15].

#### The Revised Neurophysiology of Pain Questionnaire (RNPQ)

This 12-item questionnaire was used to assess knowledge of pain neurophysiology. Responses are marked ‘yes’, ‘no’ or ‘undecided’, the latter being important to prevent respondents from guessing the answer. Scores range from 0 to 12 with high scores indicating a good knowledge of pain neurophysiology. It was found to have reasonable internal consistency (person separation index = 0.84) and intra-class correlation (ICC) = 0.97.

#### The 13-item Health Care Providers Pain and Impairment Relationship Scale (HC-PAIRS)

The modified HC-PAIRS measures attitudes towards patients with chronic pain. It is a 7-point Likert scale, with 13-items and scores range from 13 to 91 (lower scores indicate more positive attitudes towards pain). Psychometric properties of the HC-PAIRS are well established. Excellent internal consistency has been demonstrated (Cronbach’s α = 0.92) as well as good test-retest reliability [ICC = 0.84] (95% confidence interval 0.78–0.89). It is also observed to have adequate responsiveness to change [16].

#### Case vignette

Participants were asked to consider a low back pain case vignette (Supplementary file 1) to assess their pain management behaviour (clinical recommendations) towards daily activities, work, exercise and bed rest. The vignette and questions were adapted, and the number and percentage of recommendations in keeping with clinical guidelines were recorded [17].

#### Qualitative data collection

All participants were invited to attend a semi-structured focus group within a month of attending. The questions asked about their experience of PSE and how it influenced their understanding about the nature, cause and experience of pain, and their practise. During the focus group meeting the case vignette was also discussed as an aid to explore clinical reasoning processes. All interviews were undertaken by the lead author and one other author, (JM and either CR or PT), and audio recorded and transcribed verbatim by JM. Field notes were also taken to facilitate contextualisation of the recordings. It is suggested that two to three focus groups identify 80% of the relevant themes [18].

#### Data analysis

Given the exploratory nature of this study, descriptive rather than inferential statistical analysis was undertaken with quantitative data presented as mean (SD). The qualitative focus-group data was analysed using inductive reflexive thematic analysis [19]. Transcripts were read repeatedly by JM, coded and then provisionally themed using NVIVO. To ensure credibility, a second member of the research team, CR read the transcripts to ensure that codes were logical and rooted in the data. Consensus was reached on coding and then a third member of the research team DM helped to reach final consensus on the two overarching themes identified with subthemes.

## Results

Fifteen members of staff participated out of 19 eligible clinical staff members; four were nursing staff and 11 were GPs, all of whom gave informed consent to take part in this study. Of those 15 only nine completed questionnaires both pre and post intervention, five participants returned only pre-intervention surveys, and one returned only a post-intervention survey. The average age of participants was 46 years and there were 12 females and 3 males.

Pain related knowledge and attitudes were similar before and after the PSE (Table 1). Clinical recommendations before and after PSE were similar for daily activities and exercise (Table 1).

**Table 1:** Pre and post PSE data

Outcome Measure	Pre PSE (***n*** = 14)	Post PSE (***n*** = 10)
**Baseline HC-PAIRS**	34.2 (8.4)	34.2 (10.9)
**Baseline RNPQ**	8.4 (1.1)	8.8 (0.7)
**Appropriate clinical recommendations**		
**Daily activities n (%)**	8 (93)	8 (89)
**Exercise n (%)**	7 (86)	8 (89)
**Work n (%)**	9 (100)	8 (89)
**Bed rest n (%)**	7 (71)	8 (89)

Data presented as mean (standard deviation [SD]) except clinical recommendations. Clinical recommendations data presented as n = the number of appropriate recommendations and the percentage in line with clinical guidelines relating to daily activities, exercise, work and bed rest

Ten participants agreed to attend the semi-structured focus groups (six GPs and four NPs). Eight females and two males took part in two separate one hour focus groups. Two primary themes were identified. 1) difficulties of operationalising PSE principles and 2) clinician affinity for PSE but difficulty communicating it. Table 2 below summarises the qualitative findings.

**Table 2:** Themes 1 and 2 and their sub themes

***Theme 1 - Difficulties of operationalising PSE principles***	
Patients have passive and inappropriate beliefs about pain management, expecting medication and imaging	
Patients could be aggressive in pursuing these interventions	
Consultation times are limited making it ‘hard’ and a ‘fight’ to try to manage pain according to guidelines	
Difficulties of changing patient beliefs.	
Already stressful working environment with risk of litigation and comorbidities.	
Explanation of a problem is not welcomed by patients.	
Inadequate rapport with patients due to GP surgery structure and patients shopping around for the intervention they seek.	
***Theme 2 – Clinician affinity for PSE but difficulty communicating it.***	
Clinicians found the information very relevant and wanted to know more.	
Clinicians felt that some of the information was new and that some of it was a refresher of what they knew.	
The intervention gave them cause to reflect upon their management of pain.	
They wanted more information that was suitable to share with patients to help patients to understand the information and therefore accept guideline-consistent pain management.	
Limited appointment times make it harder to convey information or explain conditions like pain.	
Benefits systems perpetuate the reporting of pain.	
Clinicians sought ready ‘reels’ of information that they could convey to patients about the problem.	
Clinicians also sought other pre-prepared sources of visual and/or audio sources to explain pain to their patients.	
The PSE content was deemed to be very accessible to any audience.	

### Theme 1: difficulties of operationalising PSE principles

There were consistent perceptions that patients had very passive and inappropriate beliefs about the way their pain should be managed.

*“…many of the ones we see they don’t really, they’re not interested in the mechanism of pain… They don’t seem to be interested in physiotherapy or anything they can do themselves.”*

They almost always expected and sometimes demanded pain medication, but it was felt that such a perspective had been to some extent ‘taught’ by the medical profession.

*“…it’s to do with expectation. It’s the way we’ve taught patients over the years. If they don’t leave with a prescription, they’re not happy.”*

The expectation of patients for passive management rather than active, self-management of their pain resulted in difficult situations wherein clinicians sometimes felt that patients were *aggressive*, and they felt pressured to acquiesce to patient demands around medication.

*“…just the other week I felt really intimidated by a patient so I prescribed some Diflam for him just to get rid of him because I felt quite threatened.”*

Clinicians were aware that pain medication may not be helping the patient but they felt that the conversations they could have in the limited consultation time available made challenging a patient’s beliefs about medication ‘*hard’* and a *‘fight’*.

*“They take this painkiller and it’s actually not helping their pain, it’s helping their addiction. So it’s, so that’s the kind of message but it’s difficult in 10 minutes you know to, to chat around it and so easy thing is to just give them painkillers.”*

*“then you try to find a language to put it to the patient to see whether they will perceive it so I’ll start saying that the pain is possibly, you’re just feeding your addiction, this medication you’re taking is not actually helping your pain. They said you know I’m taking everything, maximum dose possible and I’m still in pain then you have to try to explain to them if that’s the case then why don’t you come off it. And maybe you’re just feeding your addiction.”*

Before clinicians are able to advise or direct a patient towards self-management they were having to alter existing patient beliefs about medication as noted in the quote above. Furthermore, one clinician requested that the environment in which they work was taken into context because it was a stressful one, featuring litigation, patients with mood disorders and comorbidities. Clinicians frequently termed their interactions over medication as a ‘fight’ which often led to non-guideline-consistent actions such as perpetuating prescribing or radiography.

*“For people (clinicians) who are stressed, who are already getting complaints elsewhere, to then have a conversation with someone who’s usually aggressive and difficult, that’s a big ask. And it’s not to say it’s not the right thing to do but actually to have that sort of conversation repeatedly. You know we do see a lot of chronic pain and to have that and to say no and to have to explain that and to have that fight, that’s exhausting and I think it’s easy when you’re not in the room to kind of legislate for things like this but the reality is quite different.”*

It was noted that clinician attempts to deprescribe are often not well received by patients sometimes leading to formal complaints:

*“It’s a bit like the poly-pharmacy and deprescribing. It’s not as straight forward as just taking the medication away. The number of complaints we get when pharmacists trying to be efficient remove drugs because they haven’t been used or change it over to something different, and then we get formal complaints…”*

*“…if you do that (they’ll say) I’ll buy it on the street.”*

One clinician likened the patient expectation for analgesics to the persisting expectation for antibiotics. To which one GP responded as follows:

*“Explanation for anything is not good enough.”*

An NP followed this remark with:

*“You’ve got the fight on your hands.”*

Another GP’s response summed up the likely outcome of patient demand for analgesics as follows:

*“…but it’s difficult in 10 minutes you know to, to chat around it and so easy thing is to just give them painkillers.”*

One NP participant pointed out that in order to explain things like pain, clinicians needed to build rapport with patients first and this was difficult as GPs in the surgery no longer had an assigned patient list. GPs confirmed that patients would shop around for the GP most likely to give them medication. There was one report of a ‘rare’ patient who was keen to reduce his medications.

### Theme 2 – clinician affinity for PSE but difficulty communicating it

Within this theme and linked closely to the theme above, there were subthemes including clinicians seeking more information to share with patients and the desire to learn more themselves. PSE led clinicians to think more about pain management and the factors involved.

The limitations of the ten-minute appointment were frequently referred to as well as the benefits system. Participants noted that when benefits were reassessed there was a surge in consultations for pain.

A GP referred to ‘sound bites’ and ‘reels’ he would like to learn to talk to his patients about pain. This was the HCP mode of operation when discussing any given condition, to have a set of information normally imparted. He felt that he lacked such a “reel” for persistent pain. Other clinicians referred to visuals and websites used in the intervention that they would like to access to aid patient PSE.

*“We tend to function on like a record, like if I think of contraception, there’s a reel that I use to tell patients about it but with pain I just haven’t got that yet.”*

Participants found the PSE lecture useful but a lot to digest in 70 min. A number suggested that they would like to revisit the information in “6–8 weeks” and another clinician suggested that it would take more than one session to change his practise.

All participants felt that the PSE lecture was very relevant to them, many reported that it echoed existing information and presented new information. One GP suggested that the information was highly transferrable to any audience.

*“I think the session probably has relevance to everyone, it could be a layperson, it could be a doctor, because it’s a problem and it’s just how you engage with the session isn’t it? Depending on your background you might be more into one aspect or associate more with that.”*

## Discussion

This mixed-methods, study explored HCPs experience of receiving a one-off PSE CPD session and its, impact on pain understanding and daily practise.

Clinicians demonstrated predominantly biopsychosocial understandings of pain at baseline, which were reinforced by the focus groups. However, the focus groups identified barriers to operationalising a guideline-consistent, biopsychosocial pain management approach and highlighted the strengths and limitations of the brief PSE intervention as a CPD tool.

Baseline pain knowledge and attitudes were in keeping with existing literature for GPs and nurses after an education intervention or similar to attitudes in GPs at baseline [20–22]. Furthermore, clinical vignette responses suggested that management was mostly guideline-consistent. The similar values for the outcome measures before and after PSE could be due to a ceiling effect as the results at baseline indicated relatively positive pain attitudes and pain knowledge.

Qualitative data found that the PSE intervention caused GPs and NPs to consider the obstacles to their real-life pain management. Furthermore, it led them to seek more information and aids to facilitate guideline-consistent management and operationalise their knowledge and attitudes. The prominent reason for non-guideline-consistent pain management was the breakdown of patient-centred, shared-decision-making. This was attributed to patient expectation and patients’ understanding of pain. ‘*Expectations’* for passive pain management in the form of medication reportedly dominated patient/surgery interactions relating to pain. Clinicians reported that demand for medication generally made them feel ‘*intimidated’*. This perception of patient expectation for medication and imaging is universal, increasingly prevalent and is global [23, 24].

This study identified that guideline-consistent pain management is difficult to practise in a clinic in north-east England in spite of good knowledge and attitudes amongst HCPs. Obstacles include patients’ biomedical knowledge and attitudes about pain and expectations of pain management and limited appointment times. However, if time is limited then the ethical significance to ensure that the time is of good quality is even greater [25]. Challenging patient expectation may feel uncomfortable but ‘paradoxically’ it might ‘reopen the therapeutic process’ [26].

Public understanding of pain in this large patient catchment needs to be addressed. Successful strategies have included wide reaching public health campaigns to improve health literacy amongst the public [27]. Furthermore there is a need for enhanced training for GP surgery staff to help them communicate PSE to patients as they have identified an inability to do this, which then obstructs guideline-consistent management in primary care.

### Limitations

This is a small, site-specific, exploratory study in a region where chronic pain co-exists with a diverse range of socio-economic factors therefore generalizability may be limited. However, it is an area where chronic pain is highly prevalent therefore the understanding of the challenges to good pain management in such areas is vital. The work was limited to staff working in GP practices and thus is not necessarily generalisable/transferable to other HCPs working in other contexts such as orthopaedic surgeons. Future work should be carried out to explore the impact and experience of PSE in other HCP groups. Similarly, this study only explored the impact and experience of receiving a brief PSE session delivered in a didactic fashion. The findings may have been different for a more comprehensive delivery of PSE or if PSE was delivered as part of a comprehensive CPD package for pain management.

This study is not an RCT and thus the design does not permit cause and effect analysis. Furthermore, the sample size limits quantitative inference. Future, appropriately powered multi-centred mixed methods RCTs are needed to explore the impact of PSE for GP surgery staff on both clinician behaviours and patient outcomes. Such complex interventions based upon this exploratory study could be collaboratively undertaken with stakeholders to identify optimal methods of delivering PSE to HCPs.

There was a brief dictaphone malfunction however field notes were taken throughout so that this did not result in the loss of any key information.

#### Reflexivity

Researcher background may influence data collection, analysis and interpretation. Three of the researchers (JM, CR and PG) have experience of delivering PSE and are physiotherapists who deliver it regularly to either patients or students or both.

## Conclusion

The aims of this study were to explore the impacts of a one-off PSE lecture upon HCPs at a GP surgery and the experience of receiving PSE. Knowledge and attitudes were similar before and after a brief PSE session, which may have been due to a ceiling effect. HCPs valued the PSE and it facilitated reflection on practise. Despite a preference for guideline-consistent management, there were barriers to operationalising this approach. Barriers included limited patient rapport in a large and busy surgery, limited appointment times and patients with passive, biomedical treatment expectations. Clinicians seek skills to communicate PSE and management strategies with their patients. Future large scale studies are needed to explore if similar experiences exist in different regions to confirm these exploratory findings and in other clinical subgroups.

## Supplementary Information

**Additional file 1.** Supplemental Material.

## Data Availability

The datasets used and/or analysed during the current study are available from the corresponding author on reasonable request.

## Abbreviations

CPD: Continuing professional development
GPs: General Practitioners
HC-PAIRS: Health Care Providers’ Pain and Impairment Relationship Scale
HCPs: Health Care Professionals
ICC: Intra-class correlation
NPs: Nurse Practitioners
PSE: Pain Science Education
RCTs: Randomised controlled trials
RNPQ: Revised Neurophysiology of Pain Quiz
SD: Standard deviation
