# Supplementary material for: An exploration of primary care healthcare professionals’ understanding of pain and pain management following a brief pain science education
Source: BMC Med Educ. 2022 Mar 28;22:211. doi: 10.1186/s12909-022-03265-2 (PMC8962069; doi:10.1186/s12909-022-03265-2)
Supplement: Supplementary file 1 — Additional file 1. Supplemental Material. [file 12909_2022_3265_MOESM1_ESM.docx]

**Supplemental Material**

***Case Vignette***

A 40 year-old woman presents with a six month history of low back pain. The pain came on gradually for no obvious reason. Her pain is constant with an average pain intensity of 5/10 over the past week. The patient reports moderate to high levels of disability with worse pain on prolonged standing and walking (>10 minutes). Easing factors are changing postures and lying down. She takes paracetamol as required. She feels that her activity levels have reduced and is anxious that the pain has lasted this long. There is no history of trauma. Red flag questions are negative and the neural examination is normal, suggesting that this patient has non-specific chronic low back pain. The patient has a history of Type II Diabetes, which is well controlled with medication. Otherwise, her general health is good. On physical examination, the patient reports increased pain on forward flexion and extension and her range of movement is limited in both directions. The right and left paraspinal area is tender on palpation. The patient has had one previous episode of low back pain four years ago, which resolved within three months. She had been working as a volunteer in a charity shop 2-3 mornings a week but has not done so since her back pain began. This is something she would like to return to but she is worried that this might worsen her back pain. The patient had also been attending a keep fit class twice a week but again has not attended since the onset of her back pain and is worried about the effect returning may have on her back pain.
